# Seasonal variations in circulating endocannabinoidome mediators and gut microbiota composition in humans

**DOI:** 10.1080/19490976.2025.2476563

**Published:** 2025-03-20

**Authors:** Sophie Castonguay-Paradis, Élisabeth Demers-Potvin, Gabrielle Rochefort, Sébastien Lacroix, Julie Perron, Cyril Martin, Nicolas Flamand, Frédéric Raymond, Vincenzo Di Marzo, Alain Veilleux

**Affiliations:** a Centre Nutrition, santé et société (NUTRISS), Institut sur la nutrition et les aliments fonctionnels (INAF), Québec, QC, Canada; b École de nutrition, Faculté des sciences de l’agriculture et de l’alimentation (FSAA), Université Laval, Québec, QC, Canada; c Canada Research Excellence Chair in the Microbiome-Endocannabinoidome mediators Axis in Metabolic Health (CERC-MEND), Université Laval ,Québec, Qc, Canada; d Centre de recherche de l’Institut universitaire de cardiologie et de pneumologie de Québec (IUCPQ), Québec, QC, Canada; e Département de médecine, Faculté de médecine, Université Laval, Québec, QC, Canada; f Joint International Unit on Chemical and Biomolecular Research on the Microbiome and its Impact on Metabolic Health and Nutrition (UMI-MicroMeNu) between Université Laval and Consiglio Nazionale delle Ricerche, Institute of Biomolecular Chemistry, Italy

**Keywords:** Gut microbiota, *N*-acylethanolamines, 2‑monoacylglycerol, Seasons, Vitamin D, Dietary intakes

## Abstract

**Background:**

The human gut microbiome-endocannabinoidome axis is crucial for several homeostatic processes, including inflammation and energy metabolism, and is influenced by many endogenous and exogenous factors, such as dietary habits. Changes in the gut microbiome in response to seasonal variations were previously reported and tentatively attributed to shifts in dietary patterns. However, there is a need for longitudinal studies in industrialized populations to comprehensively explore seasonal variations independently of lifestyle confounding factors.

**Objective:**

To investigate the longitudinal effects of seasonal variations on the composition of the gut microbiome and the circulating levels of endocannabinoidome mediators in humans, while elucidating the contributing factors underlying these changes.

**Methods:**

Plasma and fecal samples were collected at the end of both the winter and summer in a longitudinal cohort of 48 individuals living in Québec City (Canada). Dietary habits, medical history, fecal microbiota taxonomic composition and plasma levels of circulating *N‑*acyl‑ethanolamines (NAEs) and 2*‑*monoacyl-glycerols (2*‑*MAGs) were obtained at each time point.

**Results:**

Lower circulating levels of most NAEs were observed at the end of summer. These changes were accompanied by a reduction in the relative abundance of the *Bifidobacteriaceae* and *Lachnospiraceae* families, along with an increase in the abundance of the *Bacteroidaceae* and *Ruminococcaceae* families. These seasonal variations were not associated with concurrent changes in adiposity parameters, dietary intakes, physical activity habits, or vitamin D status. Importantly, the magnitude of the shift in gut microbiota composition from winter to summer was found to be associated with the seasonal variations in circulating endocannabinoidome (eCBome) mediators.

**Conclusion:**

This study identified specific seasonal changes in gut microbiota composition and circulating levels of several NAEs, which were not associated with vitamin D status and lifestyle habits. It underscores the importance of the gut microbiota-endocannabinoidome axis in the pathophysiology of seasonal changes, and of considering seasons in clinical trials on these systems.

## Introduction

The gut microbiota plays a major role in host metabolism, notably influencing energy metabolism, inflammation, and gut permeability.^1^ Various factors, such as environmental cues (e.g., geographical location)^2^ and lifestyle habits (e.g., dietary intake, physical activity),^3,4^ have been suggested to shape the gut microbiota in humans. Recent evidence supports the notion that the gut microbiota modulates and interacts with the endocannabinoidome (eCBome), which comprises a group of lipid mediators chemically and functionally related to the two endocannabinoids (eCBs) *N‑*arachidonoyl-ethanolamine (anandamide, AEA) and 2-arachidonoyl-glycerol (2-AG). In addition to eCBs and their main targets, the cannabinoid receptors (CB_1_ and CB_2_), the eCBome encompasses AEA and 2*‑*AG congeners, *i.e.*, the *N‑*acylethanolamines (NAEs) and 2*‑*monoacylglycerols (2-MAGs), respectively, as well as other long chain fatty acid amides.^5^ It also includes several receptors for these molecules, such as peroxisome proliferator-activated receptors (PPAR)-α/γ, G protein-coupled receptors (GPR18, GPR55, GPR110, and GPR119), and transient receptor potential vanilloid 1 (TRPV1) channels, along with the metabolic enzymes regulating the levels of the mediators. Similar to gut microbiota, the eCBome is involved in energy metabolism, adiposity regulation, and inflammation^6–8^ and has been shown to be altered by the same environmental and host determinants.^9,10^

The intricate relationship between the gut microbiome and the eCBome is highlighted by findings demonstrating that germ-free or antibiotic-treated mice exhibit altered eCBome tone in the gut, which can be reversed or attenuated by fecal microbiota transfer (FMT).^11^ Conversely, studies have shown that eCBome mediators like NAEs and 2-MAGs can directly influence fecal microbiota composition in the context of intestinal inflammation and dysmetabolism.^12,13^ Previous research has established associations between various lifestyle factors, such as diet and physical activity, which are proposed to fluctuate seasonally, and the gut microbiome-eCBome axis.^14–18^ We have reported that circulating levels of eCBome mediators are associated with total adiposity, fat distribution, physical activity and as well as recent dietary intakes of the corresponding fatty acids.^17,18^ In rodent models, the variation in ambient temperature between seasons was also found to alter gut microbiota composition.^19,20^ Previous studies in isolated communities or animals have suggested that seasonal fluctuations in gut microbiota composition are predominantly shaped by changes in dietary habits between seasons.^21–23^ Moreover, associations between vitamin D levels and the gut microbiome have also been reported,^24^ even though there is a lack of strong evidence supporting a direct link between these phenotypes.

Increased susceptibility of individuals living in higher latitudes to certain health-related seasonal changes has been documented. Daylight duration is associated with seasonal affective disorder (SAD), which manifests as depressive symptoms in winter and remits in summer.^25^ Both gut microbiota composition and circulating eCBome mediators have been linked to mental health conditions, including depression.^26^ On the other hand, cardiovascular diseases (CVD) are known to peak during winter compared to summer in countries at higher latitudes.^27^ High levels of triglycerides and lower levels of HDL cholesterol, which are associated with increased risk of CVD, are closely linked to the circulating eCBome tone, as these mediators bind and activate receptors that are key in the regulation of lipid metabolism (e.g., PPARs and CB_1_ receptors).^28,29^

Considering the potential influence of environmental factors and lifestyle habits on the gut microbiome-eCBome axis, we hypothesize that seasonal variations, such as those experienced in Québec City (Canada), may alter the composition of the gut microbiome and the circulating levels of eCBome mediators. Our aim was to determine the impact of seasonal variations on the gut microbiome-eCBome axis and to investigate whether changes in lifestyle habits, clinical parameters, or vitamin D levels account for these putative changes in individuals living in Québec City. Situated at a latitude of 46.8° N, this city experiences strong seasonal variations in temperature, ranging from an average minimum of −18°C in the winter to 25°C in the summer. Similarly, daily bright sunshine is approximately 3.3 hours in winter and 8.0 hours in summer.^30^

## Materials and methods

### Study sample

Participants were recruited at the Institute of Nutrition and Functional Foods (INAF) at Laval University in Québec City, Canada. The inclusion criteria for this study were individuals aged 18 years and above who had a basic understanding of written French. Exclusion criteria included participants previously diagnosed with enteropathies, pregnant and/or breastfeeding individuals, those consuming excessive amounts of alcohol according to recommendations (>15 drinks/week for men and >10 drinks/week for women), individuals who had taken antibiotics in the past 3 months, and those who had undergone significant weight variations (±5 kg) in the past 6 months prior to recruitment. A total of 204 participants were recruited as part of the eMECA study. Among those recruited between February and April 2018 (i.e., winter visit), 50 participants were invited to return for a second study visit planned approximately 6 months later, between August and October 2018 (i.e., the summer visit). This design was implemented to enhance the distinctions between the winter and summer seasons, thereby maximizing the potential cumulative effect of the season on the gut microbiota-eCBome axis over time. Two participants were excluded retrospectively following a review of files due to regular cannabis consumption, which may interfere with eCBome signaling. All participants provided their written informed consent. The project was approved by the Laval University Ethics Committee (2017–328) and registered to ClinicalTrials.gov registry (NCT03463304).

### Study visits

All visits were conducted at INAF’s Clinical Investigation Unit. During the first study visit in winter, participants completed medical history questionnaires and were provided with a collection kit and instructions to perform fecal collection immediately before their next visit. Additionally, they received an accelerometer to assess physical activity during the 8 to 10-day data collection period, during which participants also completed web-based questionnaires (e.g., medical history, eating habits (Three factor eating questionnaire (TFEQ) and Intuitive eating scale-2 (IES-2)), mental well-being (14-item Warwick-Edinburgh Mental Well-being Scale (WEMWBS))). After this period, participants came to INAF for a second study visit, which included anthropometric measurements and a 12-hour overnight fasting blood sampling. During the summer season, approximately 6 months after the first visit, the same data collection procedure was repeated.

### Dietary assessment

Dietary intakes were assessed using a validated self-administered web-based 24 h dietary recall (R24W).^31,32^ Participants were asked to complete three dietary recalls over 24 hours during the week preceding the fecal collection and blood sampling at INAF. One of the R24W recalls was systematically positioned on the day preceding the sampling, while the other two were randomly allocated in the week.

### Anthropometric measurements

Anthropometric measurements were conducted at the Clinical Investigation Unit by trained professionals during the second study visits. Body weight and height were measured to the nearest 0.1 kg and 0.1 cm, respectively, while waist circumference was measured to the nearest 0.1 cm. Total body fat mass and body fat distribution were assessed using a dual-energy absorptiometry scanner. (DXA, Lunar Prodigy Bone Densitometer, GE Healthcare Lunar, Madison, WI, USA) with the Lunar enCORE software version 14.1.

### Circulating endocannabinoids and related mediators

Levels of circulating endocannabinoids and of their congeners were measured in plasma. Within 30 minutes following blood collection, samples were centrifuged, aliquoted and stored at 4°C before being stored at −80°C until batch analysis. Liquid chromatography coupled with mass spectrometry (LC-MS/MS) was used to quantify plasma levels of circulating NAEs and 2 -AGs as described previously.^33^ This method allows for measuring plasma levels of several NAEs including *N-*arachidonoyl-ethanolamine (AEA), *N*palmitoyl-ethanolamine (PEA), *N-*oleoyl-ethanolamine (OEA), *N‑*linoleoyl-ethanolamine (LEA), *N‑*eicosapentaenoyl-ethanolamine (EPEA) and *N‑*docosahexaenoyl-ethanolamine (DHEA) as well as 1- and 2-monoacyl-glycerols (2-MAGs) including 2*‑*arachidonoyl-glycerol (2*‑*AG), 2-palmitoyl-glycerol (2-PG), 2-oleoyl-glycerol (2-OG), 2*‑*linoleoyl-glycerol (2-LG), 2-eicosapentaenoyl-glycerol (2*‑*EPG), 2-docosapentaenoyl-glycerol (2*‑*DPG) and 2-docosahexaenoyl-glycerol (2*‑*DHG). Monoacyl-glycerol isomers at positions 1(3) and 2 can be differentiated, but given their rapid interconversion, and the preferential esterification of polyunsaturated fatty acids (PUFAs) on the *sn*-2 position of phospholipids, 2‑MAGs derived from monounsaturated fatty acids (MUFA) and PUFA were summed up and identified as 2-MAGs.

### 16S rRNA gene sequencing

Fecal samples were collected at home by participants using the kit provided. Participants were instructed to collect fecal samples within 12 hours of the second study visit. If necessary, samples were temporarily stored in participant’s home freezer. Frozen fecal samples were brought on ice at INAF, where they were immediately stored at −20°C.

Stool bacterial DNA was extracted using the QIAamp DNA Stool Kit (QIAGEN, CA, USA) and amplification of the V3–V4 region was performed using the primers 341F (5′-CCTACG GGNGGCWGCAG-3′) and 805 R (5′-GACTACHVGGGTATCTAATCC-3′) (Illumina, CA, USA) as previously described.^34^ Briefly, libraries were purified using magnetic beads (Axygen Biosciences, CA, USA) and quality assessed (Agilent Technologies, CA, USA). High-throughput sequencing (2 × 300 bp paired-end) was performed on a MiSeq. Sequences were processed using the Dada2 package (Version 1.10.1)^35^ and associations to bacterial taxa were obtained using the Silva v132 reference database.^36^ Sequencing counts were rarefied per individual to the lowest count between the winter and summer samples to minimize the potential confounding impact of sequencing depth while maintaining the paired sample design. Sequences present in fewer than five samples were filtered out and bacterial abundances were normalized using Cumulative Sum Scaling (CSS, MetagenomeSeq R package).^37^

### Circulating25-hydroxyvitamin D

Plasma 25-hydroxyvitamin D was quantified in a single batch by an automated chemiluminescent immunoassay (Advia Centaur XPT Siemens). Measurement of 25 hydroxyvitamin D is used to determine vitamin D status, integrating both dietary, supplements and sun exposure.^38^

### Physical activity measurements

Participants wore an *ActiGraph GT3X-BT* accelerometer continuously for seven consecutive days and nights during the data collection period. Moderate to vigorous physical activity per week (MVPA) and steps per day were calculated automatically by the *ActiLife software v.6.13.3* using the Freedson (1998) equation.

### Statistical analyses

All statistical analyses were performed using R software version 4.1.1. Multiple factor analysis (MFA, *FactoMineR* R package) was performed to visualize the contribution of seasons and sex in the gut microbiota-eCBome axis.^39^ The MFA model was computed according to the following groups: gut microbiota abundances (CCS-normalized relative abundance of bacterial phyla and families in at least 10% of individuals, *n* = 27 variables), eCBome mediator levels grouped into 2MAGs (*n* = 7 variables) and NAEs (*n* = 6 variables), clinical parameters [HbA1c (%), fasting glycemia (mmol/L), fasting insulinemia (pmol/L), cholesterol (mmol/L), triglycerides (mmol/L), high-density lipoprotein (HDL) cholesterol (mmol/L), low-density lipoprotein (LDL) cholesterol (mmol/L), *n* = 8 variables] and adiposity parameters [fat mass (kg), visceral adipose tissue mass (kg) and BMI (kg/m,^2^ *n* = 3 variables]. Dietary intakes were included as macronutrient intake [fats (%), proteins (%), carbohydrates (%), fibers (g), alcohol (%), SFA (g), MUFA (g) and PUFA (g], *n* = 8 variables). The full MFA model highlights collinearity between the Phylum, Class, and Order factors, and between the Family, Genera and ASV factors. Since the Class and Order factors do not enhance the cumulative variance explained by the model, these factors were not included in the final MFA model. The Family dataset was selected over the Genera and ASV datasets based on the variance explained (AUC vs. the full model) and AIC criteria. The manuscript includes only the Phylum and Family model, while the other models yield comparable outcomes.

Relative abundance of gut microbiota taxa and circulating levels of NAEs between the winter and summer visits were compared using Wilcoxon sign rank test. Generalized linear regression models, with random effects (i.e., winter and summer visits) when specified, were employed to assess the relation between gut microbiota families, NAEs, vitamin D and MFA partial contribution (*lme4* package). Linear discriminant analysis effect size (LEfSe) analysis was conducted (*MicrobiomeMarker* package). Statistical significance was assessed using the Wilcoxon rank-sum test (*p* < 0.05). Significant features were identified using Kruskal-Wallis tests (*p* < 0.05) and Linear Discriminant Analysis score (LDA >1.5).

## Results

At recruitment, adiposity and age did not differ between men and women (Table 1). Adiposity and clinical parameters were assessed longitudinally at the first visit of the winter season, as well as at the second visit of the summer season (Table 2). On average, participants’ adiposity and metabolic parameters remained constant between the two visits, with no significant changes observed in total and visceral adiposity mass, as well as in glucose and lipid metabolism. As expected, 25-hydroxyvitamin D (Vitamin D) levels were significantly higher at the summer visit compared with the winter baseline (Table 2). Interestingly, usual dietary intakes showed relatively small variations between seasons. Notably, intakes of protein, carbohydrates, and total sugars were slightly lower in summer, but these seasonal changes were not clinically significant and were not reflected in the total energy intake (Table 3). In terms of food group intakes, only fish and seafood consumption differed between seasons (Supplementary Table S1). Eating behaviors did not differ between seasons, while mental well-being was statistically, but not clinically different (Supplementary Table S2).

**Table 1.** Participant’s characteristics at recruitment.

	Women (*n* = 32)	Men (*n* = 16)	
	Mean ± SD	Mean ± SD	*p* value
Age (years)^a^	37 ± 18	38 ± 16	NS
BMI (kg/m^2^)	24.3 ± 3.3	24.7 ± 3.3	NS
Fat mass (kg)	21.6 ± 6.9	17.4 ± 8.1	NS
Visceral adipose tissue (kg)^a^	0.3 ± 0.4	0.7 ± 0.8	NS

^a^Non-parametric Wilcoxon sign rank test.

**Table 2.** Participant’s characteristics according to visits.

	Winter visit		Summer visit			Variation
	Mean ± SD	Range	Mean ± SD	Range	*p* value	Mean ± SD
BMI (kg/m^2^)	24.4 ± 3.2	19.1–33.2	24.2 ± 3.2	19.5–33.4	0.011	−0.25 ± 0.65
Fat mass (kg)	20.2 ± 7.5	8.1–41.2	20.0 ± 7.5	8.0–40.1	NS	−0.27 ± 1.46
Visceral adipose tissue (kg)^a^	0.4 ± 0.6	0.0–2.9	0.5 ± 0.6	0.0–2.9	NS	0.02 ± 0.13
Fasting glucose (mmol/L)	4.9 ± 0.5	4.0–6.3	4.9 ± 0.4	4.3–5.9	NS	−0.03 ± 0.35
Fasting insulin (pmol/L)	9.2 ± 7.4	2.3–40.1	9.7 ± 6.1	3.6–28.8	NS	0.51 ± 4.99
HbA1c (%)	5.2 ± 0.3	4.6–6.0	5.1 ± 0.3	4.4–6.3	0.002	−0.07 ± 0.15
Triglycerides (mmol/L)	1.0 ± 0.5	0.5–3.3	1.0 ± 0.6	0.4–4.0	NS	−0.03 ± 0.50
Total cholesterol (mmol/L)	4.7 ± 1.0	3.2–7.6	4.7 ± 1.0	3.0–6.9	NS	−0.01 ± 0.67
HDL cholesterol (mmol/L)	1.7 ± 0.4	1.0–2.5	1.7 ± 0.4	1.0–2.7	NS	−0.04 ± 0.22
LDL cholesterol (mmol/L)	2.6 ± 0.9	1.3–5.5	2.6 ± 0.8	1.2–4.7	NS	0.04 ± 0.52
Vitamin D	43.6 ± 15.2	19.5–84.2	63.6 ± 15.2	30.0–97.1	0.01	20.0 ± 15.6

^1a^Non-parametric Wilcoxon sign rank test.

**Table 3.** Food intakes of the participants according to visits.

	Winter visit	Summer visit		Variation
	Mean ± SD	Mean ± SD	*p* value	Mean ± SD
Energy (kcal)	2,289.97 ± 771.14	2,136.60 ± 573.20	NS	−152.37 ± 605.04
Alcohol (g)	10.25 ± 15.44	9.45 ± 13.69	NS	−0.79 ± 18.2
Lipid (g)	87.58 ± 35.55	83.88 ± 27.79	NS	−3.71 ± 34.30
Carbohydrates (g)	279.61 ± 85.63	258.40 ± 67.59	0.044	−21.21 ± 70.96
Total sugars (g)	110.09 ± 38.32	98.93 ± 33.62	0.027	−11.16 ± 33.95
Total fiber (g)	28.94 ± 12.29	26.54 ± 9.60	NS	−2.40 ± 9.23
Protein (g)	90.30 ± 31.59	82.27 ± 25.69	0.004	−8.04 ± 18.57
SFA (g)	29.63 ± 13.51	27.33 ± 9.91	NS	−2.30 ± 12.96
SFA 16:0 (g)	15.36 ± 6.91	14.44 ± 5.41	NS	−0.92 ± 6.54
MUFA (g)	32.51 ± 13.19	30.89 ± 11.51	NS	−1.62 ± 12.70
MUFA 18:1 (g)	29.34 ± 12.46	28.06 ± 10.95	NS	−1.28 ± 12.35
PUFA (g)	17.83 ± 8.96	18.32 ± 7.63	NS	0.49 ± 9.76
PUFA 18:2 (g)	9.93 ± 6.50	9.82 ± 4.36	NS	−0.10 ± 6.98
PUFA 20:4 (g)	0.12 ± 0.08	0.13 ± 0.10	NS	0.01 ± 0.13
PUFA 20:5 (g)	0.04 ± 0.05	0.06 ± 0.09	NS	0.02 ± 0.11
PUFA 22:5 (g)^a^	0.02 ± 0.02	0.03 ± 0.04	NS	0.01 ± 0.05
PUFA 22:6 (g)	0.09 ± 0.10	0.13 ± 0.16	NS	0.04 ± 0.19

^a^Non-parametric Wilcoxon sign rank test.

### Seasonal changes in gut microbiota composition and circulating eCBome mediators

**Figure 1.**
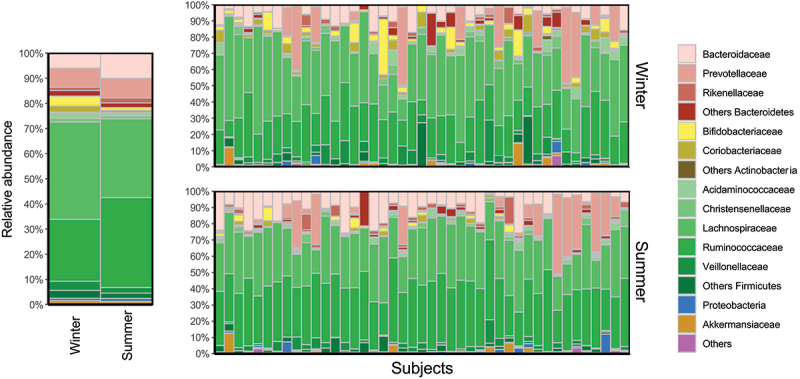
Visualization of gut microbiota family composition according to visits. Relative abundances (%) of families gut microbiota composition by season are represented as the average of the study sample (Left) and by subjects (Right).

**Figure 2.**
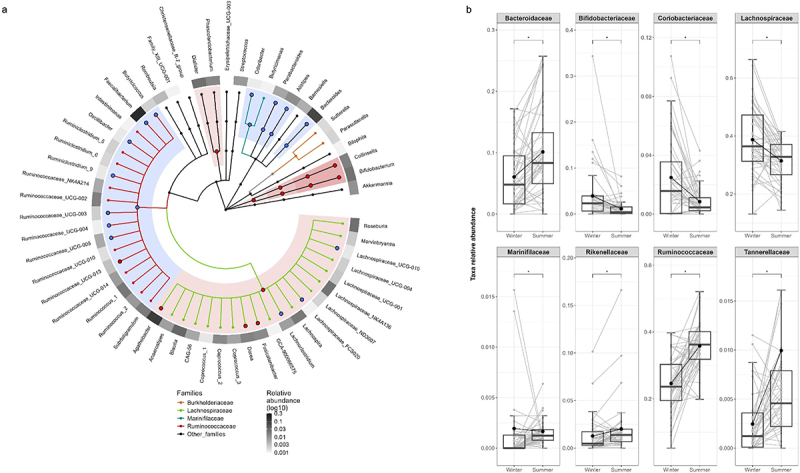
Paired-wise analysis of the gut microbiota taxonomic seasonal changes. (a) cladogram representation of the paired-wise Wilcoxon analysis showing the taxonomic ranks from the innermost phylum ring to the outermost genera ring. The outer heatmap illustrates the relative abundance of each corresponding genus. Only taxonomic ranks present in at least 60% of individuals are included. Each point represents a member within each taxonomic rank. Significant taxa (*p* < 0.05) showing increased (blue) or decreased (red) abundance between seasons are indicated by large points and shadow areas. (b) boxplots representation of significant taxonomic ranks including the median, lower/higher quartiles and 1.5× inter-quartile range whiskers. The means of the distributions are represented by a dot. *indicates significant differences between seasons using Wilcoxon sign rank test (*p* < 0.05, *n* = 48).

The Figure 1 provides histograms offering an overall view of the gut microbiota compositions of individuals by season. The taxonomic composition of the gut microbiota emerges as a discriminant factor between the winter and summer seasons for individuals in the cohort (Figure 2a). Longitudinal analyses of all taxonomic ranks in paired samples revealed that several families (i.e., *Bifidobacteriaceae, Coriobacteriacea* and *Marinifilaceae*) decreased in the summer compared to the winter, while other families (i.e., *Bacteroidaceae, Rikenellaceae, Ruminococcaceae*, and *Tannerellaceae*) increased in the summer (Figure 2a,b). Notably, a large number of genera in the *Ruminococcacea* family contribute to the increased levels of this family in the summer (e.g., *Feacalibacterium, UCG-003, UCG-004, UCG-005*), while some more abundant genera in the *Lachnospiraceae* family (e.g., *Agathobacter, Dorea, Fusicatenibacter*) contribute to the observed decrease in the summer (Figure 2a).

**Figure 3.**
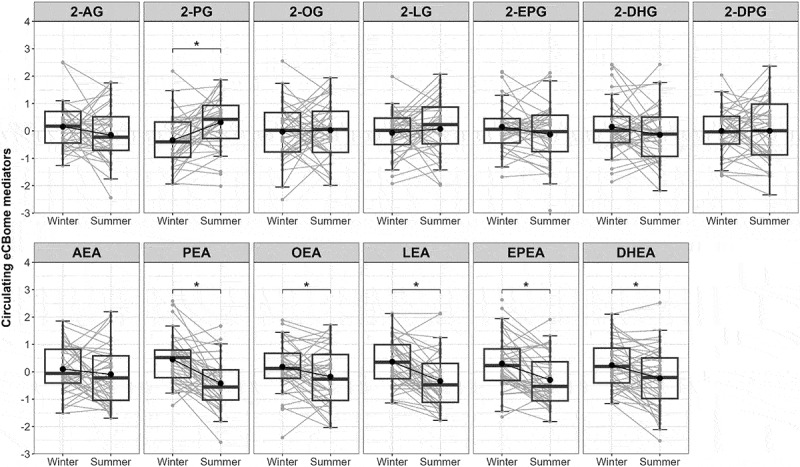
Paired-wise analysis of seasonal changes in circulating levels of eCBome mediators. Boxplots representation of 2-MAGs and NAEs including the median, lower/higher quartiles and 1.5× inter-quartile range whiskers. The means of the distributions are represented by a dot. *indicates significant differences between seasons using Wilcoxon sign rank test (*p* < 0.05, *n* = 48).

The circulating eCBome profile also plays a role in clustering individuals between winter and summer seasons, alongside gut microbiota families, independently of other confounding variables such as dietary intake, adiposity, and metabolic health. Paired longitudinal analyses reveal that the levels of all NAE congeners, except for the eCB AEA, decreased significantly during the summer compared to the winter (Figure 3). By contrast, the circulating levels of 2-MAGs remained unaffected by the seasons, except for 2-PG, which showed an increase in the summer (Figure 3).

### Gut microbiota-eCBome axis modeling with seasonal changes

A MFA model, including gut microbiota taxa as well as circulating levels of NAEs and 2‑MAGs, was computed to explore seasonal changes in the gut microbiome-eCBome axis while considering several groups of covariates (i.e., adiposity measures, clinical parameters, macronutrient intakes). Considering the collinearities between some taxonomic ranks, the cumulative variance explained by the MFA model and interpretability, only phylum and family taxa were included as factors in the final MFA model. Nevertheless, using other gut microbiota datasets to compute the MFA model provides similar outcomes.

**Figure 4.**
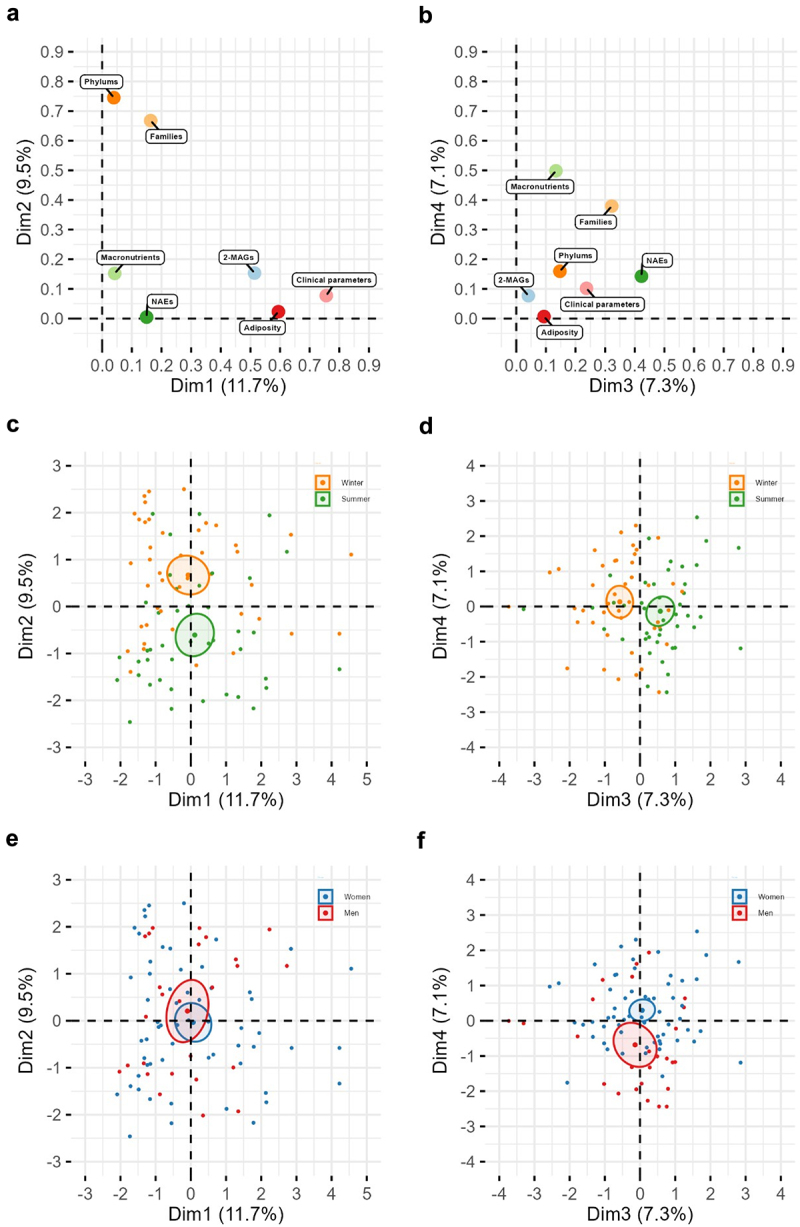
Visualization of the multiple factor analysis (MFA) modeling the gut microbiota-eCBome axis parameters with variables of adiposity, clinical parameters, dietary intakes and macronutrient intakes. Graph of factor contribution to (a) dimensions 1 and 2, and of (b) dimensions 3 and 4 of the MFA model. Graph of individuals of (c) dimensions 1 and 2, and of (d) dimensions 3 and 4 with seasons as factor variable (Winter: orange, Summer: green). Graph of individuals of (c) dimensions 1 and 2, and of (d) dimensions 3 and 4 with seasons as factor variable (women: blue, men: red). Ellipses are standard deviations from the mean center of each group of individuals.

**Figure 5.**
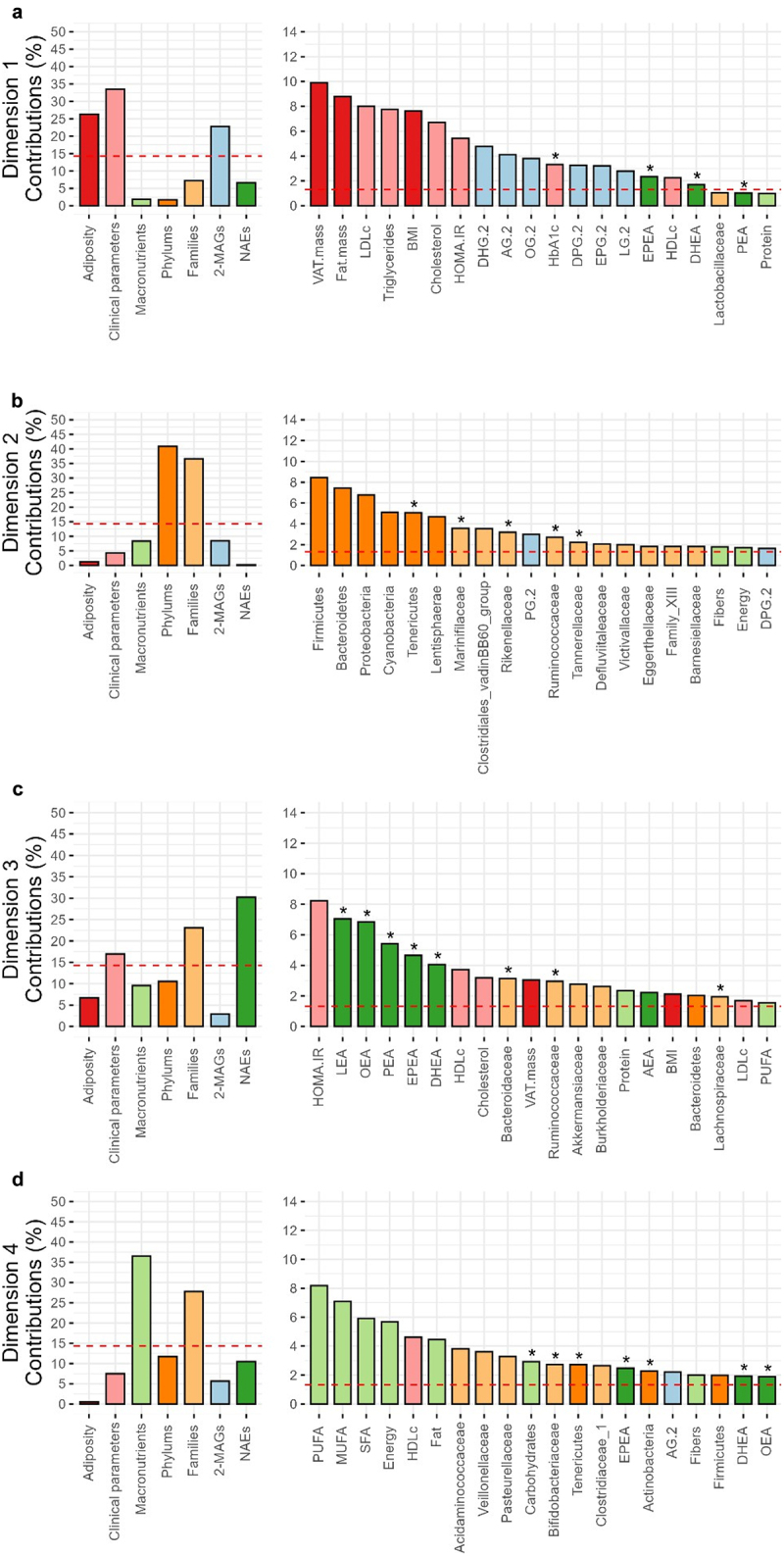
Contribution of variables of the multiple factor analysis (MFA) to (a) dimension 1, (b) dimension 2, (c) dimension 3 and (d) dimension 4. Each graph includes the contribution of each factor (Left) as well as the contribution of individual variables (Right). The dashed line corresponds to the expected value if the contribution of each variable was uniform. *indicates variables with significant differences between seasons using Wilcoxon sign rank test (*p* < 0.05, *n* = 48).

The first dimension of the model, which explained 12.0% of the variance, was mainly driven by clinical and adiposity parameters such as visceral and total adiposity, circulating triglycerides and LDLc levels, and circulating levels of 2-MAGs (Figures 4(a),5(a)) as previously reported (13). Regarding the second dimension of the MFA, explaining 9.6% of the total variance, only variables describing the composition of the gut microbiota were found to be significant (Figure 4a). Gut microbiota phyla (e.g., *Firmicutes*, *Bacteroidetes*, and *Proteobacteria*) as well as families (e.g. *Marinifilaceae*, *Clostridiales vadinBB60* group and *Rikenellaceae*) contributed to this dimension of the model (Figure 5b). Interestingly, the variables of the gut microbiota associated with this dimension enabled to distinguish individuals between the winter and the summer (Figure 4c,e). The third dimension of the MFA, which explains 7.4% of the total variance, includes the variable of circulating levels of NAEs, more specifically LEA, OEA, PEA, and EPEA, as well as the relative abundance of gut microbiota families, such as *Bacteroidaceae, Burkholderiaceae Ruminococcaceae*, and *Akkermanciaceae* (Figures 4(b),5(c)). This dimension also includes the HOMA-IR index and HDLc levels. Like the second dimension, variables of this dimension enable the clustering of individuals between the winter and the summer (Figure 4(d,f)). Finally, the fourth dimension of the model encompasses macronutrient intakes (e.g., PUFA, MUFA, SFA, energy, and % energy from fat) as well as the relative abundance of gut microbiota families (e.g., *Veillonellaceae, Pasteurellaceae* and *Acidaminococcaceae*), explaining 7.2% of the model variance (Figures 4b,5d). As expected, this dimension clusters men and women of the cohort according to dietary habits, especially total energy intakes, but not according to season (Figure 4d,f).

Overall, the second and third dimensions of the MFA model encompass several variables of the gut microbiome-eCBome axis, explaining 9.6% and 7.4% of the variance, respectively. They effectively cluster individuals based on winter and summer, independently of the contribution of other variables included in the model. This dimensional reduction will aid to thoroughly explore the relationship between seasons and the gut microbiota-eCBome axis in the subsequent analysis.

### Contribution of lifestyle habits and adiposity to gut microbiome-eCBome seasonal changes.

Considering the potential impact of seasonal variations on lifestyle factors and their relationship with the gut microbiome-eCBome axis, we explored the contribution of these parameters to the observed seasonal changes in these systems. Physical activity, measured in steps per day (Winter: 8561 ± 3543, Summer: 9075 ± 3148, NS) and moderate-to-vigorous physical activity time (Winter: 350 ± 185, Summer: 359 ± 175, NS), also remained consistent across seasons. Although there was a slight decrease in fish and seafood consumption during the summer, overall dietary intake and the proportions of fatty acid types in the diet did not differ significantly between seasons (Table 3). Additionally, carbohydrate and protein intake were slightly lower in summer compared to winter, but these differences were not clinically significant and did not affect total energy intake between seasons. Total fiber intake and the consumption of fiber-rich food groups were similar between seasons. Total and visceral fat mass, as measured by DXA, remained constant between seasons (Table 2). Consistent with these findings, adiposity and dietary intakes did not contribute to the MFA dimensions associated with seasonal changes in the gut microbiome-eCBome axis.

### Vitamin D and gut microbiota-eCBome seasonal changes

**Figure 6.**
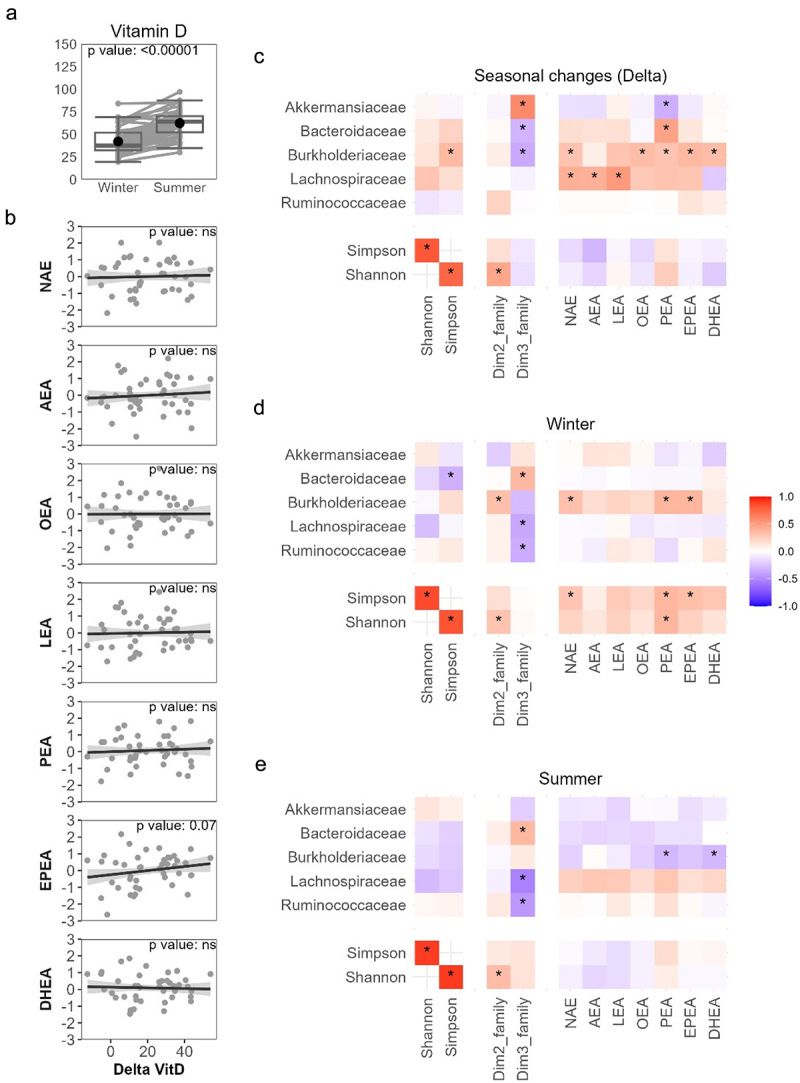
Analysis of seasonal changes in circulating levels of eCBome mediators according to the vitamin D levels. (a) boxplots representation of circulating vitamin D levels between seasons. The boxplot includes the median, lower/higher quartiles and 1.5× inter-quartile range whiskers. The means of the distributions are represented by a dot and a bold line between seasons. *indicates significant differences between clusters using Wilcoxon sign rank test (*p* < 0.05, *n* = 48). (b) linear regression analysis of seasonal changes (delta) in Z-score of NAEs and NAE congeners with circulating vitamin D levels. Heatmap of standardized regression coefficients between (c) seasonal changes (delta) in gut microbiota diversity indexes and taxa relative abundances and seasonal changes (delta) in circulating NAEs, adjusted for seasonal changes (delta) in circulating vitamin D. Heatmap of standardized regression coefficients between gut microbiota parameters in the (d) winter and (e) summer and seasonal changes (delta) in circulating NAEs, adjusted for seasonal changes (delta) in circulating vitamin D. Color intensity represents the magnitude of the standardized regression coefficients. Significant correlations following adjustments are shown (*p < 0.05; *n* = 48).

Given the pronounced seasonal variations in vitamin D status inherent to individuals living at high latitudes,^40^ such as those included in this study, it was crucial to thoroughly investigate the potential influence of this vitamin on the observed changes in circulating eCBome levels between summer and winter. As expected, circulating vitamin D levels were significantly higher at the end of the summer than the winter (Figure 6a). Remarkably, the magnitude of vitamin D seasonal changes was not associated with the seasonal changes in the circulating levels of eCBome mediators (Figure 6b). This observation, therefore, contradicts the hypothesis that vitamin D levels may drive the observed seasonal variation in the gut microbiota-eCBome axis. Except for *Lachnospiraceae*, the magnitude of vitamin D seasonal changes did not correlate with the seasonal changes in the relative abundance of gut microbiota families. Linear regression analysis between seasonal changes (delta) in gut microbiota diversity indexes and taxa relative abundances and seasonal changes (delta) in circulating NAEs, adjusted for seasonal changes (delta) in circulating vitamin D are illustrated in the heatmap (Figure 6c). Notably, the gut microbiota families identified in the previous analysis as being associated with NAE seasonal changes (specifically *Burkholderiaceae and Lachnospiraceae*, as well as *Akkermanciaceae* and *Bacteroidaceae* to a lesser extent) showed significant associations with seasonal changes in the Z-score of circulating NAEs, as well as with the NAE congeners of individuals, independent of seasonal changes in circulating vitamin D levels. Interestingly, the associations between gut microbiota taxa and circulating NAEs, after accounting for circulating vitamin D levels, were attenuated or even absent when examined in the cross-sectional data of winter and summer (Figure 6d,e). These results suggest that the seasonal changes in vitamin D levels do not play a significant role in the concomitant gut microbiota and circulating NAE seasonal changes.

### Concomitant gut microbiota and circulating NAE seasonal changes

**Figure 7.**
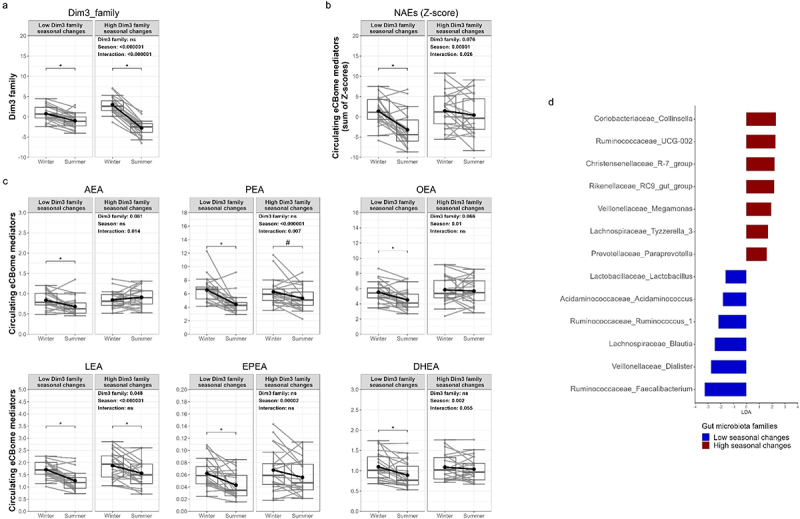
Analysis of seasonal changes in circulating levels of eCBome mediators according to gut microbiota family (dim 3) seasonal changes. Boxplots representation of (a) partial contribution of dim 3 family, (b) Z-score of NAEs and (c) NAE congeners according to the low (left) or high (right) dim 3 family groups. The boxplot includes the median, lower/higher quartiles and 1.5× inter-quartile range whiskers. The means of the distributions are represented by a dot and a bold line between seasons. Results of the General linear mixed Model (GLMM) are included within the graphs. *p < 0.05 and ^#^*p* < 0.1 indicate significant differences between seasons using Wilcoxon sign rank test (*n* = 48). (d) linear discriminant analysis effect size (LEfSe) analysis identifying microbial taxa associated with seasonal changes in the dim 3 family groups and, consequently, with the circulating NAEs seasonal changes observed (Kruskal-Wallis tests *p* < 0.05 and LDA score >1.5).

We stratified the cohort based on the seasonal changes in the gut microbiota families identified in the MFA model (Figure 7a). Individuals were categorized based on the partial contribution of gut microbiota families to the dimension 3 of the MFA model, hereafter referred to as Dim 3 family. The seasonal changes in Dim 3 family reflect the magnitude of seasonal changes in the relative abundance of families in each individual’s gut microbiota. The first group showed minimal seasonal differences in the relative abundance of the gut microbiota families *Ruminococcaceae, Lachnospiraceae Burkholderiaceae, Akkermanciaceae*, and *Bacteroidaceae* (i.e., Low Dim 3 family) compared to the second group (i.e., High Dim 3 family), which showed more pronounced changes in the relative abundance of gut microbiota families (Figure 7a). Linear Mixed Model (LMM) analysis, incorporating the interaction between seasons and Dim 3 family, revealed different seasonal responses of circulating levels of NAE congeners according to the Dim 3 family groups. It is interesting to note that the circulating NAE Z-score showed a significant decrease, specifically among individuals in the low Dim 3 with seasonally changing families (Figure 7b). Specifically, significant reductions in AEA, PEA, OEA, EPEA, and DHEA were observed solely in individuals in the low Dim 3 family group (Figure 7c). Conversely, both groups of individuals showed similar decreases in the circulating levels of LEA in summer compared to winter. These findings emphasize the intricate relationship between gut microbiota family dynamics and individual NAEs (with different receptors and functions) in response to seasonal variations.

We finally conducted a Linear Discriminant Analysis (LDA) to identify microbial taxa associated with the Dim 3 family groups and, consequently, with the seasonal changes observed in circulating NAE levels (Figure 7d). The analysis revealed several discriminant microbial taxa between the Low and High Dim 3 family groups. Within genera in the *Ruminococcaceae* family, *Faecalibacterium* and *Ruminococcus*_1 were significantly enriched in the Low Dim 3 family group, while the genus *UCG-002* was depleted in this group compared to the High Dim 3 family group. Within the genera in the *Veillonellaceae* family, *Dialister* was enriched in the Low Dim 3 family group, whereas *Megamonas* was depleted in this group. Within the genera in the *Lachnospiraceae* family, *Blautia* was enriched in the Low Dim 3 family group, whereas the genus *Tyzzerellacea* was depleted in this group. Additionally, genera such as *Acidaminococcus* and *Lactobacillus* were enriched in the Low Dim 3 family group, while genera including *Collinsella*, *RC9*, *R-7*, and *Paraprevotella* were enriched in the High Dim 3 family group. Overall, the LDA results provide valuable insights into the composition of the gut microbiota associated with the seasonal changes in the circulating levels of NAEs.

## Discussion

The present study aimed to assess the influence of seasonal variations on the gut microbiome-eCBome axis and to identify environmental factors contributing to the putative changes observed between summer and winter. Our findings reveal seasonal shifts in gut microbiota composition alongside alterations in eCBome mediator profiles. Specifically, we observed a significant decrease in circulating levels of NAEs, particularly PEA, LEA, EPEA, and DHEA, from winter to summer in healthy individuals residing in Québec City (Canada). In addition, we uncovered several longitudinal changes in the relative abundance of specific gut microbiota taxa between these two seasons. Interestingly, these seasonal variations were not explained by concurrent changes in adiposity parameters, dietary intakes, or physical activity habits, all of which are known to strongly influence the eCBome.^9,10^ Moreover, despite significant interindividual seasonal differences in vitamin D levels within the population, individuals with larger increases in vitamin D levels did not exhibit greater responses in the eCBome between seasons. Notably, our analysis identified specific seasonal changes in gut microbiota composition that were associated with the alterations observed in the circulating levels of several NAE congeners, and these associations were independent of vitamin D status, adiposity, metabolic parameters, and dietary intakes, which otherwise are also known to affect the gut microflora.

The impact of circannual rhythms on the gut microbiome and circulating eCBome has received limited attention to date. A study conducted in an American Hutterites cohort revealed an increase in the relative abundance of *Bacteroidetes* during summer compared to winter, which was associated with higher consumption of fruit and vegetables during the summer season.^22^ Previous research, including both human and animal studies, has suggested that seasonal variations in the gut microbiota are largely influenced by changes in dietary habits between seasons.^21–23^ Notably, these studies have primarily been conducted in cohorts of tribes or isolated communities (e.g., Hutterites or Hadza), which show strong adaptations in eating patterns that are more strictly dependent on seasonally available products than in populations living in industrialized countries.^21,22^ While our study includes only one sampling per season over a single year and cannot definitively conclude on the recurrence of these seasonal variations, such annual patterns in gut microbiota have been previously demonstrated. Indeed, Smits et al. revealed a cyclic seasonal pattern in the gut microbiome of Hadza hunter-gatherers of Tanzania, in which taxonomic changes recur in the subsequent season.^21^

The study cohort included in this analysis did not exhibit notable changes in measures of adiposity and usual dietary intake. Although statistically significant differences in dietary intakes were observed between seasons, these variances were deemed clinically trivial. For example, the variation in carbohydrates between seasons was less than 15 g, which holds low significance in clinical practice. Additionally, while there was a slight increase in fish and seafood consumption during summer, overall dietary intake, and the relative proportions of fatty acids in the diet remained largely constant between seasons. This apparent stability in food intake across seasons may be attributed to the accessibility and availability of produce year-round, particularly in populations where agriculture and food chains have been industrialized.^41^ Even isolated communities, such as the Inuit from the Northern Québec (Canada), who historically had a highly seasonal diet, now have year-round access to industrialized foods, leading to more stable gut microbiota compositions than their counterparts in the south of the province.^42^ Another possibility is that the dietary assessment tools used did not capture the diversity of some food items.^43^ It could be hypothesized that food diversity would better reflect seasonal variations in dietary habits than macronutrients and food groups. Moreover, higher food diversity during the summer season may be associated with the observed increased diversity in the gut microbiota during this season. For example, while the total fiber intake and the consumption of fiber-rich food groups such as whole grains, vegetables, and legumes were similar between seasons, slight changes in food items may have had an impact on the nature and proportion of fibers in the diet between the summer and winter seasons. Similarly, the same amounts of fruit and vegetables may not reflect the diversity of these items, as well as their nutrient contents, such as polyphenols, known to modify the composition of the gut microbiota.^44^ Another potential confounding factor could be the food preparation methods (e.g., fresh or cooked), since thermal processing of food has been shown to negatively impact gut microbiota diversity in mice.^45^

The findings of our study suggest that the reported seasonal changes in the gut microbiome-eCBome axis were not explained by changes in physical activity either. We did not observe significant changes in physical activity time and intensity between seasons in our cohort. Previous studies have shown that Canadians tend to engage in greater amounts of physical activity during the summer season, whereas we did not find evidence of any variation.^46^ It is important to emphasize that our study participants were likely to be more active than the general population, with an average of almost 10,000 steps per day for each season. It is possible that more active individuals remain active regardless of environmental changes. Unfortunately, to the best of our knowledge, none of the studies examining seasonal changes in gut microbiota have included physical activity as a confounding parameter.

Contradictory findings have been reported in the literature in relation with the specificity of seasonal influence on various responses by geographical factors such as light exposure and ambient temperature. Indeed, significant shifts in the levels of circulating eCBome mediators, including 2-AG, AEA, PEA, and OEA, have been observed during hibernation in mammals,^47–49^ but little evidence exists regarding such variations in humans. However, a recent study demonstrated that skin exposure to Ultraviolet B (UVB) light in humans with low 25 (OH) D serum levels leads to increased alpha diversity of the gut microbiota and enrichment in several families, including *Clostridiales vadinBB60* group, *Desulfobacteraceae, Ruminococcaceae*, and *Rikenellaceae*,^50^ which align with the higher abundance of these families observed in the summer in our study. Nonetheless, our study also revealed lower levels of *Lachnospiraceae, Coriobacteriaceae*, and *Marinifilaceae* in the summer. Low intensity UVB light elevates 2-AG and PEA levels and reduces AEA levels in human keratinocytes cocultured with melanocytes.^51^ While this suggests that UVB exposure could be a potential determinant of the eCBome, our results do not support this conclusion. In fact, we observed a decrease in the levels of almost all NAEs in the summer, contrary to what would be expected based on the association with UVB exposure. Another study in mice reported a significant shift in microbiota composition following exposure to cold temperatures.^52^ Consistent with these findings, the changes observed in the microbiota composition in our study may also be partly attributed to marked temperature changes between seasons. However, it is important to acknowledge that individuals living in a cold climate, such as those in this study sample, are not constantly and directly exposed to cold temperatures due to clothing and housing adaptations. It is essential to note that light exposure, particularly UV exposure, and temperature are among several other factors associated with seasonal variations. Therefore, longer periods spent indoors and other changes in habits may represent more important contributors to the seasonal variations observed here.

Further studies are required to determine any potential causal relationship between seasons and circulating levels of NAEs. Interestingly, we observed that the winter season is associated with elevated levels of lipid mediators known to activate a variety of receptors with beneficial effects (e.g., GPR55, GPR119, GPR110, PPARs) on metabolism, intestinal permeability, and low grade inflammation.^53–55^ This seasonal alteration may thus represent an innate adaptive response to conditions (reduction of physical activity, higher consumption of high calorie foods) that, although not observed in our cohort, are likely to lead to metabolic dysregulation. However, the lack of comprehensive knowledge on the role of NAEs in the context of seasonal variations, prevent us from forming a definitive understanding of the physiopathological implications of the seasonal changes in circulating eCBome signaling observed here.

Considering the susceptibility of individuals living in higher latitudes to certain health-related seasonal changes (e.g., seasonal affective disorder (SAD)^25^ and cardiovascular diseases (CVD),^27^ seasonal alterations in the gut microbiota-eCBome axis may be relevant to these conditions. While the hypotheses regarding the implication of the gut microbiota-eCBome axis in seasonal metabolic changes could not be confirmed using our relatively healthy cohort, the results presented here suggest avenues for future investigation into this emerging area of research.

In conclusion, the findings of this study suggest that humans experience seasonal shifts in their gut microbiota composition that are accompanied by, and possibly related to, changes in their circulating eCBome profile, and are independent of adiposity, dietary intakes, physical activity and vitamin D levels. These findings underscore the importance of accounting for temporal variations in future clinical trials assessing gut microbiota composition and eCBome mediator levels. Moreover, they emphasize the necessity of investigating the role of the gut microbiota-eCBome axis in various metabolic and psychological diseases across different seasons.

## Supplementary Material

Supplemental Material

## Data Availability

Data availability Sequencing data for the 16S rRNA sequences were deposited in the NCBI GenBank under BioProject ID PRJNA644138 and under SRA accession number SUB7687442. Individual de-identified subject data, including a data dictionary, related to the analyses included in this manuscript, will be made available from the corresponding author on a reasonable request.
