# Supplementary material for: Seasonal variations in circulating endocannabinoidome mediators and gut microbiota composition in humans
Source: Gut Microbes. 2025 Mar 20;17(1):2476563. doi: 10.1080/19490976.2025.2476563 (PMC11926903; doi:10.1080/19490976.2025.2476563)
Supplement: Supplemental Material [file KGMI_A_2476563_SM3234.docx]

## SUPPLEMENTARY MATERIALS

**Supplementary table 1.** Dietary group intakes according to visits.

|  | **Winter visit** | **Summer visit** |  |
| --- | --- | --- | --- |
|  | **Mean ± SD** | **Mean ± SD** | ***p* value** |
| **Whole grains** | 2.2 ± 1.8 | 1.9 ± 1.7 | NS |
| **Refined grains** | 3.1 ± 1.9 | 3.3 ± 1.9 | NS |
| **Vegetables** | 3.4 ± 1.9 | 3.9 ± 2.1 | NS |
| **Fruits** | 2.3 ± 1.5 | 2.2 ± 1.3 | NS |
| **Fruit juice1** | 0.5 ± 0.8 | 0.4 ± 0.7 | NS |
| **Legumes, nuts and seeds** | 1.0 ± 0.9 | 0.8 ± 0.7 | NS |
| **Milk and substitutes** | 2.4 ± 1.5 | 2.0 ± 0.7 | NS |
| **Olives and olive oil1** | 0.5 ± 0.8 | 0.5 ± 0.6 | NS |
| **Fish and sea food1** | 0.2 ± 0.2 | 0.3 ± 0.4 | 0.04 |
| **Poultry** | 0.3 ± 0.4 | 0.3 ± 0.4 | NS |
| **Eggs** | 0.6 ± 0.5 | 0.5 ± 0.7 | NS |
| **Sweets** | 1.0 ± 0.8 | 0.7 ± 0.7 | 0.07 |
| **Red meats and charcuteries** | 0.7 ± 0.6 | 0.5 ± 0.8 | NS |

^1^Non-parametric Wilcoxon sign rank test. Values are expressed in portions.

**Supplementary table 2.** Mental well-being and eating behaviours scores according to visits.

|  | **Winter visit** | **Summer visit** |  |
| --- | --- | --- | --- |
|  | **Mean ± SD** | **Mean ± SD** | ***p* value** |
| Warwick-Edinburgh Mental Well-being Scale^1^ | 54.3 ± 7.7 | 55.6 ± 7.4 | 0.04 |
| Intuitive eating scale-2 total score | 3.6 ± 0.5 | 3.6 ± 0.5 | NS |
| Unconditional permission to eat^1^ | 3.4 ± 0.7 | 3.4 ± 0.6 | NS |
| Eating for physical rather than emotional reasons | 3.7 ± 0.9 | 3.7 ± 0.8 | NS |
| Reliance on hunger and satiety cues | 3.5 ± 0.7 | 3.6 ± 0.7 | NS |
| Body-food choices congruence^1^ | 4.2 ± 0.7 | 4.1 ± 0.8 | NS |
| Cognitive restraint | 7.3 ± 4.1 | 7.8 ± 4.2 | NS |
| Disinhibition | 5.2 ± 2.9 | 5.0 ± 2.4 | NS |
| Susceptibility to hunger^1^ | 4.0 ± 2.5 | 4.1 ± 2.6 | NS |

^1^Non-parametric Wilcoxon sign rank test
